# Single-cell transcriptome revealed dysregulated RNA-binding protein expression patterns and functions in human ankylosing spondylitis

**DOI:** 10.3389/fmed.2024.1369341

**Published:** 2024-05-06

**Authors:** Zheng Ren, Chenyang Li, Jing Wang, Jiangtao Sui, Yuan Ma

**Affiliations:** ^1^Xinjiang Institute of Spinal Surgery, Sixth Affiliated Hospital of Xinjiang Medical University, Ürümqi, Xinjiang, China; ^2^Microsurgery Unit, The Third People’s Hospital of Xinjiang, Ürümqi, Xinjiang, China

**Keywords:** Ankylosing Spondylitis(AS), RNA-binding proteins (RBPs), Single-cell RNA sequencing (scRNA-seq), B cells, CD8^+^ T cells, Gene

## Abstract

**Objective:**

To explore the expression characteristics and regulatory patterns of RBPs in different immune cell types of AS, and to clarify the potential key role of RBPs in the occurrence and development of AS disease.

**Methods:**

PBMC sample data from scRNA-seq (HC*29, AS*10) and bulk RNA-seq (NC*3, AS*5) were selected for correlation analysis.

**Results:**

(1) Compared with the HC group, the numbers of B, DC (dendritic cells), CD14^+^ Mono and CD8^+^ T cells were increased in AS group, while the numbers of platelet (platelets), CD8^+^ NKT, CD16^+^ Mono (non-classical monocytes), Native CD4^+^ T and NK were decreased. (2) Through the analysis of RBP genes in B cells, some RBPs were found to play an important role in B cell differentiation and function, such as *DDX3X*, *SFPQ*, *SRRM1*, *UPF2*. (3) It may be related to B-cell receptor, IgA immunity, NOD-like receptor and other signaling pathways; Through the analysis of RBP genes in CD8^+^ T cells, some RBPs that play an important role in the immune regulation of CD8^+^ T were found, such as *EIF2S3*, *EIF4B*, *HSPA5*, *MSL3*, *PABPC1* and *SRSF7*; It may be related to T cell receptor, TNF, IL17 and other signaling pathways. (4) Based on bulk RNA-seq, it was found that compared with HC and AS patients, differentially expressed variable splicing genes (RASGs) may play an important role in the occurrence and development of AS by participating in transcriptional regulation, protein phosphorylation and ubiquitination, DNA replication, angiogenesis, intracellular signal transduction and other related pathways.

**Conclusion:**

RBPs has specific expression characteristics in different immune cell types of AS patients, and has important regulatory functions. Its abnormal expression and regulation may be closely related to the occurrence and development of AS.

## 1 Introduction

Ankylosing spondylitis (AS) is an inflammatory rheumatic disease that affects the axial bones and joints and is characterized by abnormal immune response and bone remodeling (1, 2). The prevalence rate is 0.09%–0.3% (3, 4). Patients suffer from persistent pain, spinal stiffness, fatigue, and progressive damage to spinal structure and function, affecting quality of life, and may lose the ability to work if left untreated (5, 6). Immunoinflammatory response is considered to be an important factor in the pathogenesis of AS. There are multiple immune cell number and function abnormalities in PBMC in AS patients. For example, the proportion of Th17 cells increased (7), the activation of CD8^+^ T cells was abnormal (8), and the proportion of Treg cells decreased (9). Plasmacytoid dendritic cells (pDC) in peripheral blood can, on the one hand, secrete inflammatory cytokines (TNF-α, IL-6, and IL-23) (10). On the other hand, the expanded CD4^+^ T cells can be induced to differentiate into pro-inflammatory Th17 cells, producing IL-17A and TNF-α (11). At the same time, the proportion of B lymphocytes in AS patients was higher than that in healthy controls (12). Therefore, controlling AS immune inflammation and adjusting immune homeostasis are key to treating and even delaying disease progression in the early stages. It can be seen that the study of immune cells in the occurrence and development of AS may be a breakthrough point to further understand the pathogenic mechanism of the disease and study targeted drugs. However, relevant studies are still scarce. RNA-binding proteins (RBPs) are key effectors of gene expression (13), and their abnormal expression is the basis of many diseases (14, 15), including AS diseases (16–18). However, there are few studies on the expression patterns of RBP in different immune cells of AS and the abnormal regulation related to AS disease.

In recent years, single-cell transcriptome sequencing has provided a technical means to reveal the overall level of gene expression in a single cell and reflect the inter-cell heterogeneity (19–21). There have been some studies using single-cell RNA sequencing (scRNA-seq) to study the specific gene expression profile information of specific cells during the occurrence and development of AS (22–24). In-depth analysis of these single-cell transcription datasets contributes to understanding the pathogenesis of disease and is a rich resource for developing novel targeted therapeutic strategies. Based on published single-cell data, we aim to reveal the expression characteristics and regulatory patterns of RBPs in different immune cell types of AS, and further elucidate the potential key role of RBPs in the occurrence and development of AS diseases.

## 2 Materials and methods

### 2.1 Analysis of scRNA-seq data

#### 2.1.1 Retrieval and process of public data

Unique molecular identifier (UMI) count matrix of single-cell RNA-seq data of 10 AS (ankylosing spondylitis), and 29 HC (healthy control) were downloaded from GSE194315. The UMI count matrix was converted into a Seurat object by the R package Seurat (25) (version 4.3.0). Cells with UMI numbers < 500 or with detected genes < 200 or with over 15% mitochondrial-derived UMI counts were considered low-quality cells and were removed. When quality control screens out some low-quality cells, we screen out genes expressed by a few cells (<5).

#### 2.1.2 scRNA-seq data preprocessing

After quality control, the UMI count matrix was log normalized. The top 2,000 variable genes with the highest degree of variation were used to create potential anchors with Seurat’s FindIntegrationAnchors function. Subsequently, IntegrateData function was used to integrate data. To reduce the dimensionality of the snRNA-Seq dataset, principal component analysis (PCA) was performed on an integrated data matrix. With Elbowplot function of Seurat, top 50 PCs were used to perform the downstream analysis. The main cell clusters were identified with the FindClusters function offered by Seurat, with resolution set as default (res = 0.6). Finally, cells were clustered into 10 major cell types. And then they were visualized with tSNE or UMAP plots. To identify the cell type for each cluster, we detected gene markers for each cell clusters using the “FindMarkers” function in Seurat package (v4.3.0), then we annotated cell types using ScType tools (26) with previously published marker genes (27).

#### 2.1.3 Differential gene expression analysis

Differentially expressed genes (DEGs) were determined with the FindMarkers / FindAllMarkers function from the Seurat package (one-tailed Wilcoxon rank sum test, pvalues adjusted for multiple testing using the Bonferroni correction). For computing DEGs, all genes were probed that the expression difference on a natural log scale was at least 0.2 and adjusted *p*-value was less than 0.05.

### 2.2 RNA-binding protein regulatory program analysis

1. Firstly, a catalog of a catalog of 2,141 RNA-binding proteins (RBPs) retrieved from four previous reports (28–31). Co-expression associations of RBPs and targeted genes were built by the “grn” algorithm from the SCENIC python workflow (version 1.12) using default parameters.^1^ Networks of the modules with RBPs and their target genes were visualized by Cytoscape (v3.9.1).^2^
2. Retrieval and process of bulk RNA-seq data: Public expression matrix data files of bulk RNA-seq of peripheral blood tissue lines (GSE181364, https://www.ncbi.nlm.nih.gov/geo/query/acc.cgi?acc=GSE181364) were downloaded from the National Center for Biotechnology Information (NCBI).
3. Differentially expressed gene (DEG) analysis: The expression levels of genes were evaluated using FPKM. The software DEseq2, which is specifically used to analyze the differential expression of genes, was applied to screen the raw count data for DEGs. The results were analyzed based on the fold change (FC ≥ 1.2or ≤ 1/1.2) and *P*-value (*P*-value ≤ 0.05) to determine whether a gene was differentially expressed. Then expression profile of differentially expressed RBPs were filtered out from all DEGs according a catalogue of a catalog of 410 RNA-binding proteins (RBPs) retrieved from B cells and CD8^+^ T cells.
4. Alternative splicing analysis: Regulatory alternative splicing events (RAS) were defined and quantified using the SUVA (v2.0) pipeline. The different splicing of each group was analyzed. Reads proportion of SUVA AS event (pSAR) of each AS events were calculated.
5. Correlation analysis: Co-expression analysis was performed for all differentially expressed RBP and RAS. Meanwhile, Pearson correlation coefficient between differentially expressed RBP and RAS was calculated, and DERBP-RAS relationship pairs satisfying absolute value of correlation coefficient ≥ 0.7 and *p*-value ≤ 0.05 were screened.
6. Functional enrichment analysis: To sort out functional categories of genes, Gene Ontology (GO) terms and KEGG pathways were identified using KOBAS 2.0 (32). Hypergeometric test and Benjamini-Hochberg FDR controlling procedure were used to define the enrichment of each term.

## 3 Results

### 3.1 scRNA-seq analysis of human PBMC from healthy donors and as patients identified distinct cell types

**Figure 1:**
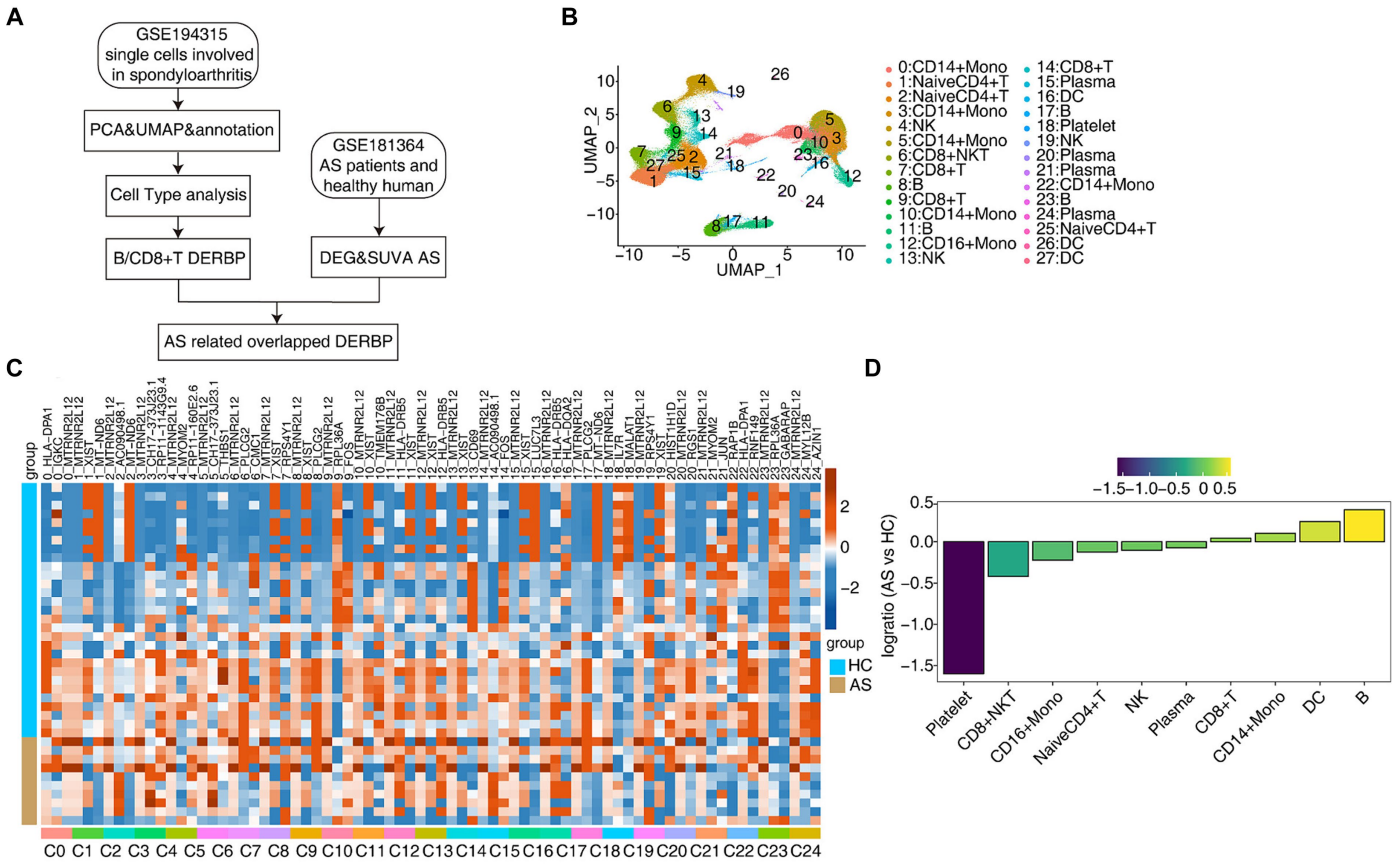
scRNA-seq analysis of human PBMC from healthy donors and AS patients identified distinct cell types. **(A)** Flow chart for analysis of the scRNA-seq and bulk RNA-seq. **(B)** UMAP plot of single-cell transcriptomic profiles from ankylosing spondylitis patients and healthy subjects. Colors indicate cell clusters along with annotations. **(C)** DEG heatmap. **(D)** Rank order based on decreasing values of the relative frequency ratio between two sample groups.

In order to further explore the key regulatory factors specific to PBMC cells in AS patients, we collected the single-cell transcriptome (scRNA-seq) data of 15 published PBMCs (33), including healthy people (HC*5) and AS patients (AS*10), and also used the traditional bulk RNA-seq data for integrated analysis (Figure 1A). Normalization of the expression matrix of scRNA-seq was carried out, dimensionality reduction of principal components was analyzed, and the first 50 principal components were selected for UMAP reduction and visualization. After unbiased clustering analysis, 28 clusters were obtained (Figure 1B). Using the newly published sctype software combined with the cell marker gene in the paper (33), we identified 10 different cell types (Figure 1B). Key marker genes in different cell types were shown (Supplementary Figures S1A,B). Next, we analyzed the proportion of each cell subtype in each sample group, the main cell types being CD14^+^ Mono (monocyte), Naive CD4^+^ T (initial CD4^+^ T cells), CD8^+^ T (effector CD8^+^ T cells), B (B lymphocytes), NK (natural killer cells), CD8^+^ NKT (natural killer like CD8^+^ T cells; Figures 1C,D). These results present a panoramic picture of changes in the composition and proportion of different immune cells in the PBMC of patients with AS and healthy individuals.

### 3.2 scRNA-seq analysis revealed dysregulated RBPs and their regulatory functions in B cells

**Figure 2:**
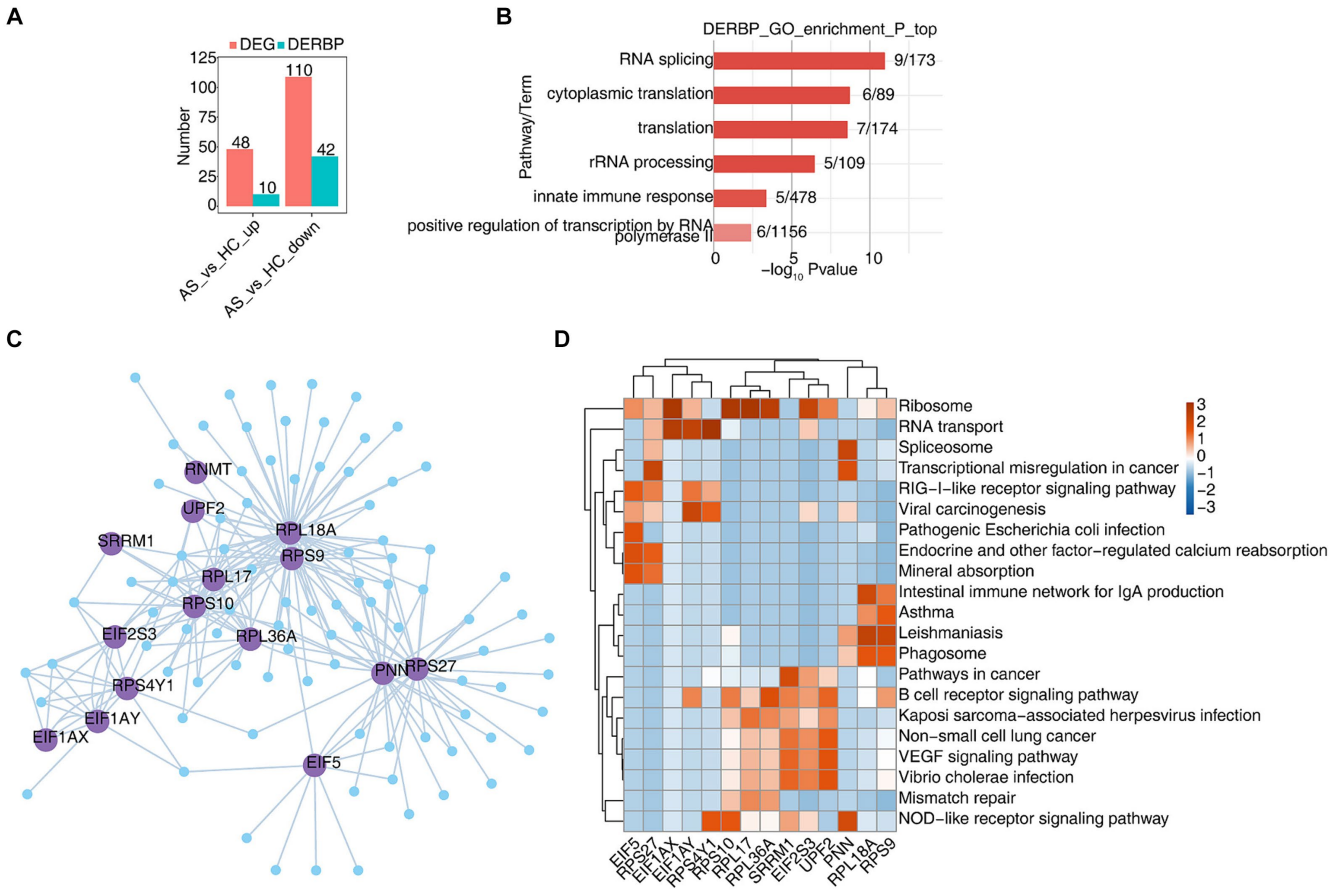
scRNA-seq analysis revealed dysregulated RBPs and their regulatory functions in B cells. **(A)** Bar graphs show the number of DEGs and DERBPs. **(B)** Bar plot showing the most enriched GO results of DERBPs. **(C)** Cytoscape shows the co-expression network comprising differentially expressed RBPs from B cells. Edges connect RBP-target gene pairs while nodes represent DEGs. RBPs are displayed in larger font size and deep purple color. **(D)** Gene ontology enrichment analysis of KEGG pathway of target genes for each RBP from graph **(C)**.

B cells exert humoral immune function by secreting antibodies after antigen stimulation. In order to explore the function of RBP in B cells, we first analyzed differentially expressed genes (DEGs) in B cells using scRNA-seq data, and found that 48 genes were upregulated, 10 of which were RBPs, and 110 genes were downregulated, 42 of which were RBPs (Figure 2A). The heat map showed the expression levels of differentially expressed RBP genes in AS and HC groups. Some RBPs, such as *DDX3X* , *SFPQ* , *SRRM1* , *UPF2* , etc. play an important role in B-cell-related diseases (Supplementary Figure S2A) (34–37). Functional cluster analysis was performed on 52 differentially expressed RBP genes, and it was found that they were mainly concentrated in RNA processing, variable splicing, translation and other related pathways (Figure 2B). To further confirm the regulatory function of differential RBPs on gene expression changes, we extracted the differentially expressed RBPs whose gene functions are enriched in relevant pathways such as RNA stability, transport and translation, and used the pySCENIC grn tool to predict the coexpressed genes of these RBPs. RBP-target gene pairs with importance > 60 were screened according to the obtained importance value, and then Cytoscape was used to map the co-expression network composed of RBPs and their target genes (Figure 2C). KEGG functional cluster analysis of these RBPS-regulated target genes showed that the target genes of *EIF1AY* , *RPS10* , *RPL36A* , *SRRM1* , *EIF2S3* , **UPF2*,* and *RPS9* were enriched in the B-cell receptor signaling pathway. Target genes of *RPL18A* and *RPS9* are enriched in IgA producing intestinal immune networks, phagosomes, and some immune disease pathways. The target genes of *RPS4Y1* , *RPS10* , *SRRM1* and *PNN* are enriched in the NOD-like receptor signaling pathway (Figure 2D). Supplementary Figures S2B–E show the expression of *SRRM1* , *UPF2* , *EIF2S3* , and *RPS10* genes in all B cells of different samples.

### 3.3 scRNA-seq analysis revealed specific RBPs regulatory module in CD8^+^ T cells

**Figure 3:**
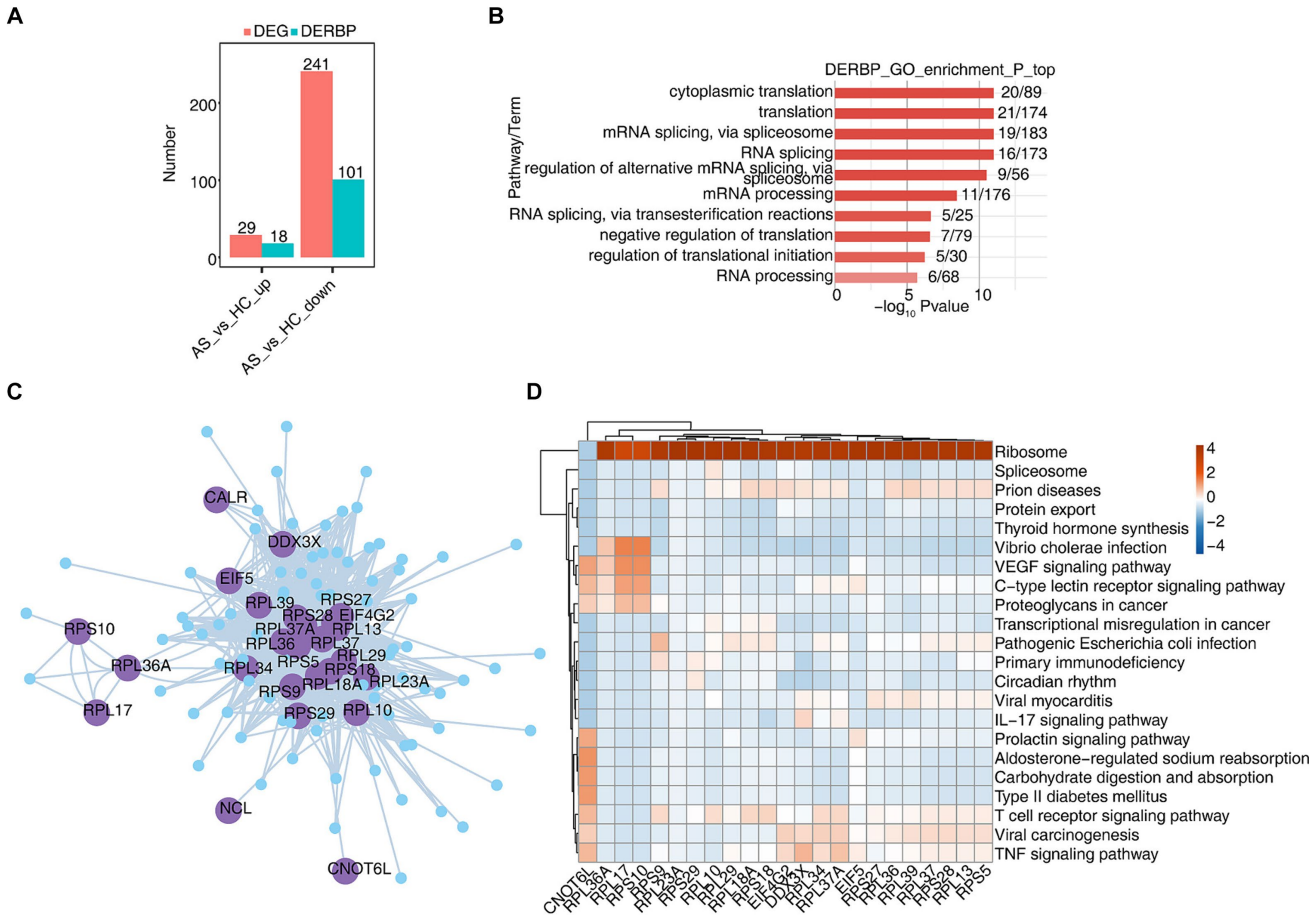
scRNA-seq analysis revealed specific RBPs regulatory module in CD8^+^ T cells. **(A)** Bar graphs show the number of DEGs and DERBPs. **(B)** Bar plot showing the most enriched GO results of DERBPs. **(C)** Cytoscape shows the co-expression network comprising differentially expressed RBPs from CD8^+^ T cells. Edges connect RBP-target gene pairs while nodes represent DEGs. RBPs are displayed in larger font size and deep purple color. **(D)** Gene ontology enrichment analysis of KEGG pathway of target genes for each RBP from graph **(C)**.

CD8^+^ T cells generally refer to cytotoxic T lymphocytes, which are specific killer T cells and can secrete various cytokines to participate in immune function. In order to analyze the function of RBPs in CD8^+^ T cells of AS and HC, we first analyzed differentially expressed genes (DEGs), and found that there were 29 upregulated genes, of which 18 were RBPs, and 241 downregulated genes. Of these, 101 are RBPs and 44% of DEGs are RBPs (Figure 3A). The heat map showed the expression levels of differentially expressed RBP genes in AS and HC groups. Some RBPs, such as *EIF2S3*, *EIF4B*, *HSPA5*, *MSL3*, *PABPC1,* and *SRSF7*, play an important role in CD8^+^ T immune regulation (Supplementary Figure S3A) (38–41). Functional cluster analysis was performed on 119 differentially expressed RBP genes, and it was found that they were mainly concentrated in RNA processing, variable splicing, transport, and translation related pathways (Figure 3B). To further confirm the regulatory function of differential RBP on gene expression changes, we extracted differentially expressed RBPs with gene function enrichment in RNA stability, transport and translation-related pathways, and used pySCENIC grn tool to predict these RBPs co-expression genes. RBP-target gene pairs with importance > 60 were screened according to the obtained importance value, and then Cytoscape was used to map the co-expression network composed of RBPS and their target genes (Figure 3C). KEGG functional cluster analysis of these RBPS-regulated target genes showed that the target genes of *CNOT6L*, *RPS9*, *RPL10*, *RPL18A*, *RPL34,* and *RPL37A* were enriched in the T cell receptor signaling pathway. The target genes of *CNOT6L*, *EIF4G2*, *DDX3X*, *RPL34*, *RPL37A,* and *RPS25* were enriched in the TNF signaling pathway. The target genes of *DDX3X* and *RPL37A* are enriched in the IL17 signaling pathway (Figure 3D). Supplementary Figures S3B–E show the expression of *CNOT6L*, *DDX3X*, *EIF4G2*, and *RPL18A* genes in all CD8^+^ T cells of different samples. These results indicate that the expression of RBPs in CD8^+^ T cells changes during the development of AS disease, which may be related to T cell receptor, TNF, IL17 and other signaling pathways.

### 3.4 Integration with bulk RNA-seq reveals that extensive alternative splicing regulation in ankylosing spondylitis may be related to the abnormal regulation of RBPs

**Figure 4:**
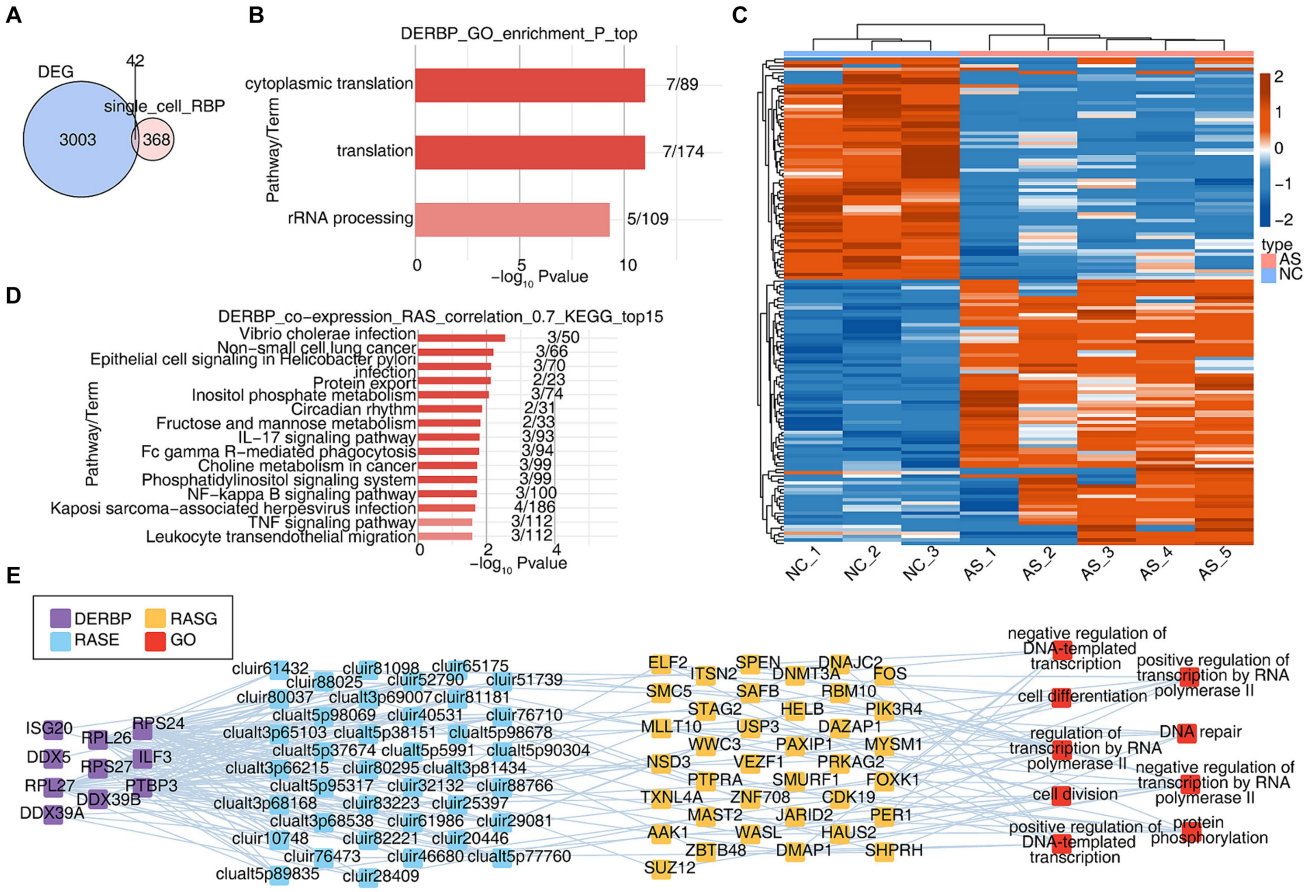
Integration with bulk RNA-seq reveals that extensive alternative splicing regulation in ankylosing spondylitis may be related to the abnormal regulation of RBPs. **(A)** The Veen shows the number of DERBPs between the DEG and DERBP of single cell. **(B)** Bar plot showing the most enriched GO results of DERBPs. **(C)** The Heatmap showing the splicing ratio of specific RAS (PSAR ≥ 50%) in the AS vs. NC group. **(D)** Bar plot showing the top 15 most enriched KEGG results of specific DERBP co-expressed by specific RAS. **(E)** Co-expression analysis of specific DERBP and specific RAS of key GO biological process results. Cutoffs of *P*-value ≤ 0.05 and Pearson coefficient ≥ 0.7 or ≤−0.7 were applied to identify the co-expression pairs. The network showing the co-expressed GO pathway for specific DERBP and specific RAS.

Cell-specific RBPs are involved in regulatory processes critical to the expression of cell-specific functional proteins (e.g., alternative splicing and translation control of mRNA). We wanted to know about the potential post-transcriptional regulatory role of RBP in AS, particularly on variable splicing. Due to the inability to obtain complete variable splicing information from single-cell data based on the 10X platform, we obtained bulk RNA seq data of PBMCs from healthy and AS patients from the SRA database and conducted a systematic analysis. Firstly, differentially expressed genes were analyzed, with a total of 3,045 differentially expressed genes (DEGs). The overlapping analysis of these DEGs and the differentially expressed RBP genes (DERBPs) of all cells in the single-cell data was conducted, and 42 overlapping genes were found (Figure 4A). GO analysis was conducted on the functions of these 42 overlapping RBP genes, and it was found that they were enriched in variable splicing and translation related pathways (Figure 4B). Since variable splicing is known to be highly complex in humans and mice, we used the newly published SUVA software to analyze the variable splicing of RNA-seq data in NC group (healthy people) and AS group (patients with ankylosing spondylitis). Comparison between groups identified hundreds or even thousands of regulated differential splicing events (RASEs; Supplementary Figures S4A–C). PCA analysis based on the splicing ratio of these RASEs can clearly separate the two groups of samples, indicating that the RNA variable splicing landscape of the NC and AS groups has undergone great changes (Supplementary Figure S4D). The heat map shows the splicing ratio of RASEs in the NC and AS groups (Figure 4C). The genes of these RASEs are enriched in transcriptional regulation, protein phosphorylation and ubiquitination, DNA replication, angiogenesis, intracellular signal transduction and other related pathways (Supplementary Figure S4E). Further, we used the overlaps of differentially expressed RBPs and RASEs in bulk RNA-seq and scRNA-seq to conduct co-change analysis to predict the potential regulatory role of RBPs on variable splicing. After screening (|correlation| ≥ 0.95, *p*-value ≤ 0.01), GO functional analysis of RASEs significantly associated with these RBPs enriched in pathways related to transcriptional regulation, protein phosphorylation, DNA replication and cell differentiation (Figure 4E). KEGG function analysis of RASEs co-expressed with RBPs was concentrated in IL17 signaling pathway, FcγR-mediated phagocytosis, NF-κB signaling pathway, TNF signaling pathway, and pathways related to leukocyte transendothelial migration (Figure 4D). Supplementary Figure S4F heat map shows the expression of these RBPs in each sample (Supplementary Figure S4F). These results suggest that the differential expression of RBPS-regulated variable splicing genes in PBMC immune cells of AS patients may be related to inflammation and immune activation pathways, thereby regulating immune cell phenotype and function during the occurrence and development of AS.

## 4 Discussion

Immune inflammatory response is considered to be an important factor in the pathogenesis of AS, in which the destruction of immune homeostasis is closely related to AS. A large number of studies have confirmed that there are smooth dysregulation of the secretion of pro-inflammatory/anti-inflammatory factors in the pathogenesis of AS, inducing inflammatory factor storms, and the migration of inflammation to the ligament attachment site, causing pain and stiffness (42–44). Effecting CD4^+^ T cells are the main part of immune response. CD4^+^ T cells are divided into Th1, Th2, Th17, Treg and other cell subsets, secreting a variety of cytokines and participating in the occurrence of various physiological and pathological mechanisms. Stimulated by pro-inflammatory cytokines, the downstream JAK2/STAT3 signaling pathway is activated to promote the expression of RORγt, resulting in the specific differentiation of CD4^+^ T cells to Th17 cells and promoting the occurrence of AS inflammation (45, 46). At the same time, IL23 binds to IL23R and continues to maintain Th17 differentiation, expansion and function (47). In PBMC patients with AS, the ratio of Th17 was increased, transcription factor RORγt was highly expressed, and JAK2/STAT3 was activated (7). Some researchers have proposed that AS is an autoimmune disease driven by CD8^+^ T cells activated by antigen peptide HLA-B27. However, no significant activation of CD8^+^ T cells was observed in patients with AS (8). Although HLA-B27 transgenic rats showed AS phenotype, However, the clearance of CD8^+^ T cells does not improve the disease (48, 49). Our experiments showed that CD8^+^ T cells were significantly increased in AS patients compared with normal people, so the role of CD8^+^ T cells in AS remains elusive. Foxp3-expressing Treg cells, also known as suppressive T cells, exist naturally in the immune system and play an indispensable role in dominating autoimmune tolerance and maintaining immune balance in the body. Treg cells can be produced in the thymus (called tTreg cells) and peripherally (called pTreg cells). The relationship between Treg cells and AS has attracted more and more attention, and some preliminary studies have been conducted. However, some results presented conflicting points. For example, some studies showed that the proportion of Treg cells in peripheral blood of AS patients was not significantly different from that of healthy controls (50), while other literatures showed that the proportion of Treg cells in AS patients was significantly higher than that of healthy controls (51). Some studies even showed that the proportion of Treg cells in peripheral blood of patients with AS decreased (9). Multiple subgroups of T cells are related to each other in development and function, and the cytokines secreted by them constitute the cellular network that regulates immunity.

After encountering antigen stimulation, B cells further differentiate into plasma cells with the help of Tfh cells, and exert humoral immunity function by secreting antibodies. In the past, B cells have received relatively little attention in the pathogenesis of AS, mainly due to the lack of disease-defining autoantibodies, and there is increasing evidence that B cells are involved in AS. Some studies have shown that the proportion of B lymphocytes in patients with active AS is significantly higher than that in patients with stable AS and healthy controls (12). However, another study showed that the proportion of CD27^+^ B cells in B lymphocyte subsets of AS patients decreased, and the CD86^+^ and CD27^−^ CD95^+^ B lymphocyte subsets increased compared with healthy controls (52). Our study found that the proportion of B cells in the AS group was higher than that in the HC group, which was consistent with the results of Lin et al. (12).

Dendritic cells (DC) are an antigen-presenting cell and a very important class of native immune cells that can activate primary T cells. The direct interaction of DC with HLA-B27 may be the initial factor leading to the development of crista arthritis, and then DC migration and initiation of IL-17-activated inflammatory response and osteogenesis (53). Peripheral pDC was isolated from controls of AS patients, and it was found that the expression of pDC transport molecules CCR6 and CCL20 was enhanced in AS patients, and the secretion of several inflammatory cytokines (including TNF-α, IL-6, and IL-23) by pDC was significantly increased (10). In the spinal arthritis model of HLA-B27 transgenic rats, CD4^+^ T cells with DC-induced amplification exhibited pro-inflammatory Th17 phenotype, producing IL-17A and TNF-*α* (11). Consistent, our study found that the proportion of DC increased in the AS group. Therefore, DC plays an important role in the regulation of inflammation and immune response in AS.

RBPs interact with RNA to form ribonucleoprotein (RNP) complexes that regulate the maturation and fate of target RNA substrates and regulate many aspects of gene expression, including mRNA splicing, cutting, and polyadenylation, RNA stability, RNA localization, RNA editing, and translation, among others (31). RBPs can recognize hundreds of transcripts and form an extensive regulatory network that helps maintain cell homeostasia. Notably, cell-specific RBPs are involved in regulating the expression of a variety of cell-specific functional proteins, affecting some vital biological processes. For example, the expression of *YTHDF2* mRNA in PBMC may be involved in the pathogenesis of AS, and the prediction model based on *YTHDF2* can be used as a marker for disease diagnosis and progression (16). m^6^A modification produced by *METTL14* in the 3 ‘UTR region of the *ELMO1* gene in AS patients promotes gene expression, while inhibition of *ELMO1* expression in mouse models produces therapeutic effects (17). Six genes in the MHC Class III region, *DDX39B*, *DXO*, *LSM2*, *NELFE*, *PRRC2A*, and *SKIV2L*, encode RBPs and have a well-defined role in post-transcriptional gene regulation and RNA monitoring, and these genes may have important functions in immunity and be associated with autoimmune diseases (18). A recent study found that relative RBPs expression is different between B cells and T cells, and differentiation into effector and memory cells alters RBPs expression, resulting in preferential expression of different classes of RBPs (38).

Similarly, our study identified alterations in the expression of RBPs within B cells and CD8^+^ T cells throughout the progression of AS. Specifically, aberrant expression of *DDX3X*, *SFPQ*, *SRRM1*, and *UPF2* genes was observed in B cells of AS patients, suggesting their potential involvement in B-cell development, processing, and translation pathways. Moreover, through predicting the co-expression target genes of these RBPs, we unveiled pivotal roles of *EIF1AY*, *RPS10*, *RPL36A*, *SRRM1*, *EIF2S3*, *UPF2,* and *RPS9* target genes in the B-cell receptor signaling pathway, while *RPL18A* and *RPS9* target genes were implicated in the generation of intestinal IgA immunity. Additionally, phagosomes were implicated in certain immune disorders, with *RPS4Y1*, *RPS10*, *SRRM1*, and *PNN* target genes playing roles in the NOD-like receptor signaling pathway. Furthermore, abnormal expression of *EIF2S3*, *EIF4B*, *HSPA5*, *MSL3*, *PABPC1*, and *SRSF7* genes was noted in CD8^+^ T cells of AS patients, potentially influencing T cell development, processing, and translation pathways. Predicted co-expression target genes of these RBPs revealed the involvement of *CNOT6L*, *RPS9*, *RPL10*, *RPL18A*, *RPL34*, and *RPL37A* target genes in the T cell receptor signaling pathway. Additionally, *CNOT6L*, *EIF4G2*, *DDX3X*, *RPL34*, and *CNOT6L* target genes were implicated in the TNF signaling pathway, while *DDX3X* and *RPL37A* target genes played key roles in the IL17 signaling pathway.

Through bulk RNA-seq data analysis, differential splicing genes and their functional pathways in AS were studied, and it was found that transcriptional regulation, protein phosphorylation and ubiquitination, intracellular signal transduction and other related pathways played an important role in the occurrence and development of AS. Through the combined analysis of single cell data and bulk RNA-seq, some RBPs with important regulatory roles in AS were predicted and screened, and the potential variable splicing regulatory functions of these RBPs were analyzed. In this study, it was found that *DDX3X* expression was abnormal in patients with AS. Yong Dai et al. used a combination of single-cell analysis, transferase accessible chromatin sequencing (scATAC-seq) and single-cell RNA sequencing (scRNA-seq) to explore the key genes associated with the pathogenesis of AS and their related mechanisms. Eighteen cell types were identified from peripheral blood mononuclear cells of AS patients and normal controls, and it was found that the pathogenic gene NF-κB involved in the progression of AS was derived from CD8^+^ T cells. In addition, abnormal tumor TNF pathways mediated by abnormal expressions of TNF, NF-κB, FOS, JUN, and JUNB were observed, and scATAC-seq results confirmed the presence of abnormal accessibility binding sites for the transcription factors FOS, JUN, and JUNB. The possible mechanism by which NF-κB normally regulates FOS, JUN and JUNB to promote AS progression was revealed (23). Studies have shown that upregulation of *DDX3X* protein can increase the expression of nuclear NF-κB related proteins and reduce the inflammatory response of OA (54), which may also occur in AS process. This study found abnormal expression of *SFPQ* in patients with AS, and previous studies have found that regulating the SFPQ-AKT-RUNx2 pathway can reduce chondrocyte injury and the progression of osteoarthritis (55). It is speculated that *SFPQ* may also have such a role in the course of AS disease. Stimulated by pro-inflammatory cytokines, the downstream JAK2/STAT3 signaling pathway is activated, which will promote AS inflammation (41, 42). *SRRM1* affects the levels of p-JAK2, JAK2, p-STAT3 and STAT3, key molecules in the JAK/STAT pathway, thereby promoting inflammation (56). The abnormal expression of *SRRM1* in B cells of AS patients is found, which may promote the occurrence of AS inflammation through the activation of JAK/STAT pathway. Further experimental studies are needed. Heat shock protein family A member 5 (*HSPA5*) is a recently discovered ferritin inhibitor. Previous studies have shown that knocking on *SND1* upregulates *HSPA5* and *GPX4* in rat cartilage, which can inhibit inflammatory damage and alleviate OA progression (57). Also in the study of osteoporosis, *in vitro* studies showed that *HSPA5* negatively regulated osteogenic differentiation, while inhibitor *HA15* enhanced osteogenic differentiation. The abnormal expression of *HSPA5* in AS patients may be due to the effect of T cells on inflammatory injury, formation and differentiation of pathological bone. It was found that inhibition of *PABPC1* improved the mRNA stability of *SOX9* and inhibited the degradation of ECM induced by inflammation, thereby reducing the progression of OA (58). Inhibition of *PABPC1* may slow down the occurrence and development of AS inflammation, thus becoming a potential therapeutic target. Serum/arginine-rich shear factor 7 (*SRSF7*) belongs to proteins in the shear factor family that are involved in multiple aspects of RNA processing, including shear, mRNA nucleation, mRNA stability, and translation. Has been shown to play a carcinogenic role in a variety of cancers. *SRSF7* has been found to be a novel modulator of m6A that promotes m6A methylation near the binding site of mRNA involved in cell proliferation and migration by recruiting methyltransferase complexes (59). Whether *SRSF7* can regulate the proliferation and migration of inflammatory cells and cytokines in the process of AS inflammation by regulating m^6^A needs to be further explored.

There is a certain relationship between intestinal IgA and AS, and studies have shown that the pathogenesis of AS is closely related to the disturbance of intestinal flora (60). Intestinal lgA is an important part of intestinal mucosal immune system, which can protect the body from pathogens. In the case of intestinal flora disorder, some microorganisms such as Klebsiella, Salmonella and Yersinia may stimulate the intestinal mucosa to produce too much IgA, and then trigger abnormal immune response and inflammation. These abnormal immune responses may lead to damage and inflammation of the joint tissue, which may induce or aggravate the symptoms of AS (61). NF-κB signaling is a key mediator of cytokine and chemokine transcription, and is divided into “canonical NF-κB signaling pathway” and “non-canonical NF-κB signaling pathway.” Among them, non-canonical NF-κB signaling pathway is more important for intestinal IgA production (62). The non-canonical pathway involves activation of NF-κB inducing kinase (NIK), which leads to proteolytic processing of NF-κB2 to p52 (63). Studies have shown that intestinal epithelial NIK is essential for M cell maintenance and initiation of protective IgA response, and mice lacking IgA B cells lacking *Jh^−/−^* are highly susceptible to DSS induced colitis. In patients with ulcerative colitis, a strong increase in epithelial NIK signaling was observed (62). Our study found that the *RPL18A* gene plays a role in intestinal immunity that produces IgA. The ribosomal protein L18a synthesized by the *RPL18A* gene is a member of the ribosomal protein and plays an important role in protein synthesis. We speculated that L18a might regulate epithelial M cells through non-canonical NF-κB signaling pathway, leading to IgA production in B cells, thereby affecting the occurrence and development of AS. First, we can establish a model of *RPL18A^+/−^* epithelial M cells to observe the expression of NF-κB2 in M cells after *RPL18A* changes. Secondly, the production of IgA in M cells was detected after the use of NF-κB activator/inhibitor, and the relationship between IgA and NF-κB2 and B cell activity was analyzed. Then M cells and B cells were co-cultured to observe the response of B cells to IgA produced by M cells and the functional changes of B cells. Finally, a mouse model of AS was established to investigate the effects of *RPL18A*^+/−^ on spinal inflammation, NF-κB and intestinal IgA in AS mice. A series of experiments verified that *RPL18A* affected the occurrence and development of AS by regulating the production of IgA by epithelial M cells through non-canonical NF-κB signaling pathway.

This study is based on sequencing and analysis of AS and HC samples of PBMC samples from published single-cell data. We know that there can be heterogeneity of cells in different tissues within an organism. Next, we need to collect spinal tissue samples from clinically healthy people and AS patients, and then analyze whether the expression characteristics of RBPs in different immune cell types in AS patients and healthy people are consistent with the above results through single-cell sequencing, and verify these differential expression of RBPs and its potential regulation of DEGs through cell modeling *in vitro* and animal models.

In conclusion, we found that some RBPs play an important role in the pathogenesis and development of AS by influencing the phenotype and function of immune cells by participating in the IL17 signaling pathway, FcγR mediated phagocytosis, NF-κB signaling pathway, TNF signaling pathway and leukocyte transendothelial migration. In the future, the continuous in-depth study of these RBP may find new detection markers and drug targets for AS.

## Data availability statement

The authors acknowledge that the data presented in this study must be deposited and made publicly available in an acceptable repository, prior to publication. Frontiers cannot accept a manuscript that does not adhere to our open data policies. https://www.jianguoyun.com/p/DTbDwW0QuoOpDBidvbEFIAA.

## Ethics statement

The studies involving humans were approved by the Ethics Committee of the Sixth Affiliated Hospital of Xinjiang Medical University. The studies were conducted in accordance with the local legislation and institutional requirements. Written informed consent for participation was not required from the participants or the participants’ legal guardians/next of kin in accordance with the national legislation and institutional requirements. The manuscript presents research on animals that do not require ethical approval for their study.

## Author contributions

ZR: Writing – original draft, Writing – review & editing. CL: Writing – original draft, Writing – review & editing. JW: Writing – original draft, Writing – review & editing. JS: Data curation, Formal analysis, Methodology, Supervision, Validation, Writing – review & editing. YM: Conceptualization, Methodology, Project administration, Resources, Supervision, Validation, Writing – review & editing.

## Footnotes

^1^ http://scenic.aertslab.org

^2^ https://cytoscape.org/
