# Supplementary material for: Single-cell transcriptome revealed dysregulated RNA-binding protein expression patterns and functions in human ankylosing spondylitis
Source: Front Med (Lausanne). 2024 May 6;11:1369341. doi: 10.3389/fmed.2024.1369341 (PMC11104332; doi:10.3389/fmed.2024.1369341)
Supplement: Supplementary file 2 [file Data_Sheet_2.docx]

Supplementary Material

# Supplementary Figures and Tables

## Supplementary Figures


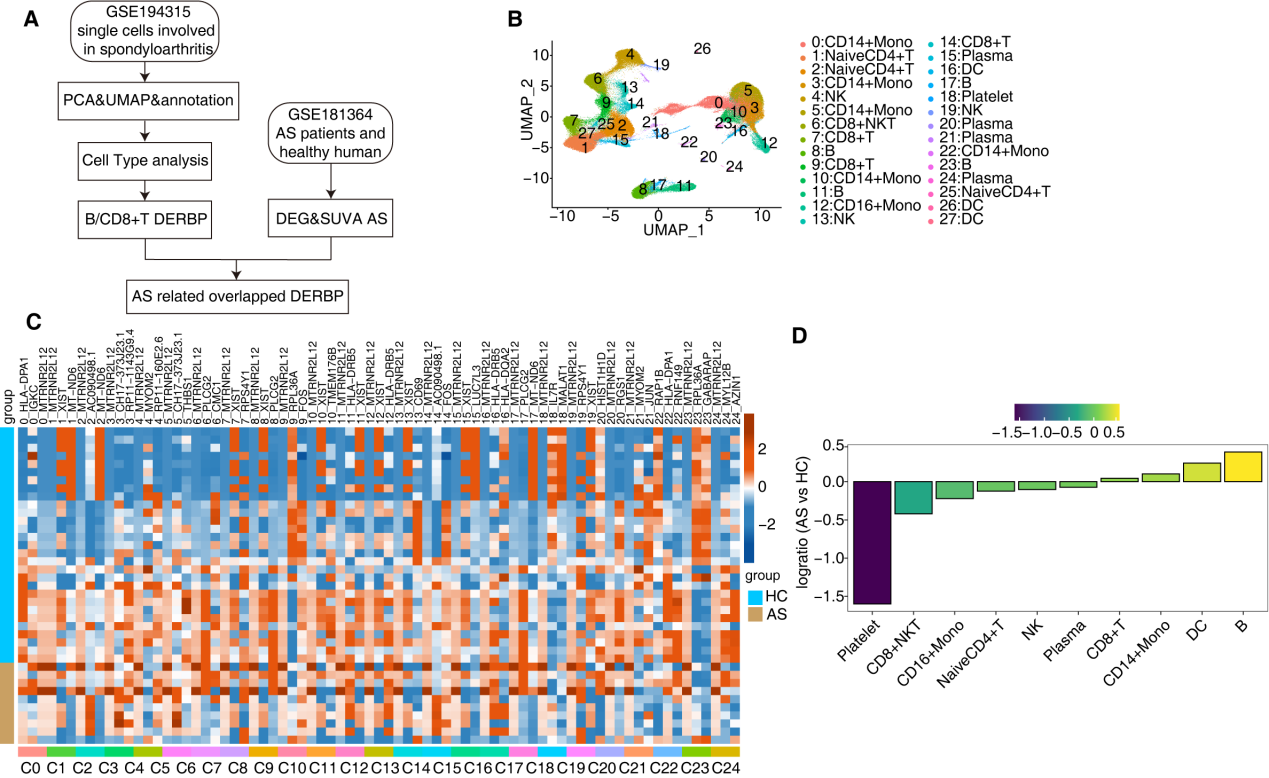


**Supplementary Figure 1.** **ScRNA-seq analysis of human PBMC from healthy donors and AS patients identified distinct cell types.**

1. Flow chart for analysis of the scRNA-seq and bulk RNA-seq.
2. UMAP plot of single-cell transcriptomic profiles from ankylosing spondylitis patients and healthy subjects. Colors indicate cell clusters along with annotations.
3. DEG heatmap
4. Rank order based on decreasing values of the relative frequency ratio between two sample groups.

**
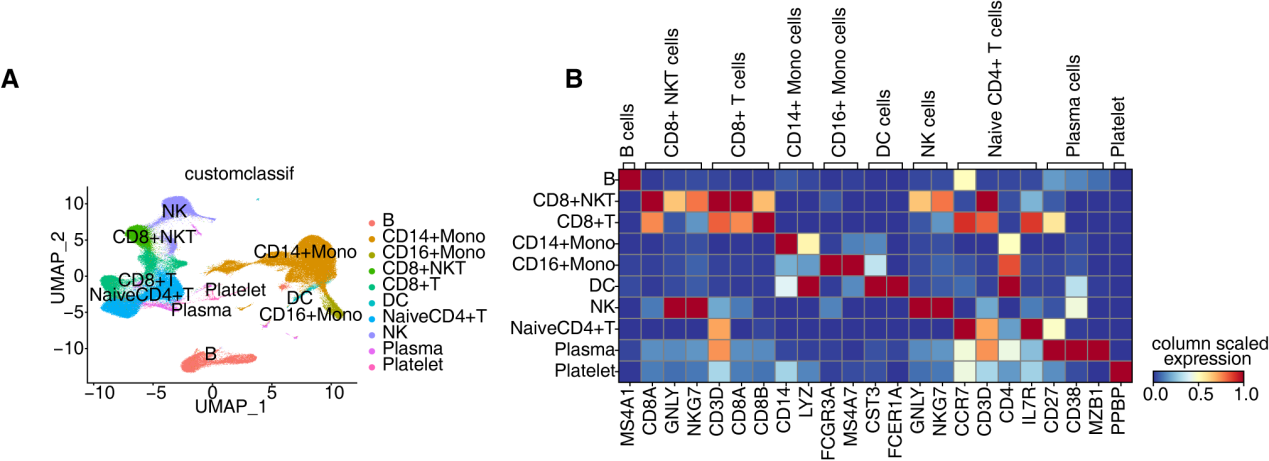
**

**Supplementary Figure S1.ScRNA-seq analysis of human PBMC from healthy donors and AS patients identified distinct cell types.**

1. UMAP of 10 different cell types. Colors represent cell clusters and comments.
2. Dot plot showing expression of representative markers of ankylosing spondylitisin each cell type.

**
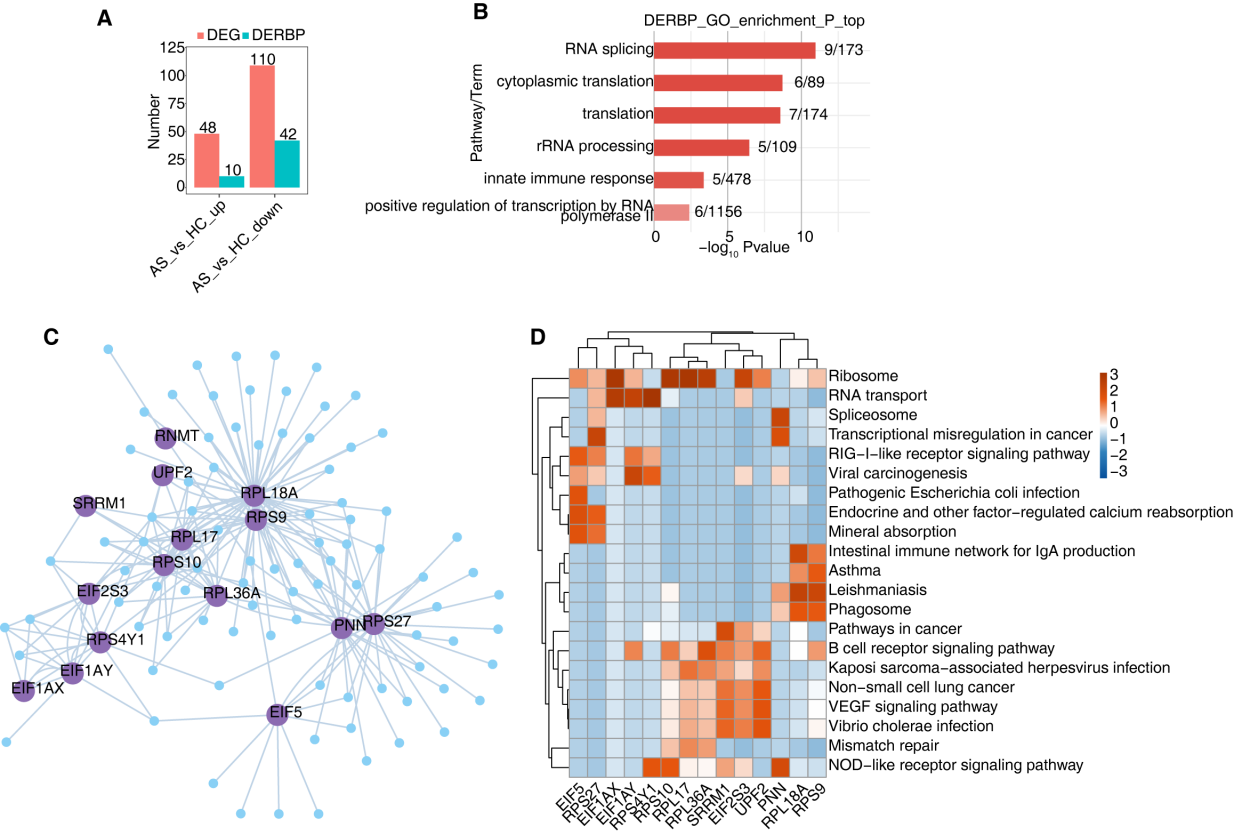
**

**Supplementary Figure 2. scRNA-seq analysis revealed dysregulated RBPs and their regulatory functions in B cells.**

1. Bar graphs show the number of DEGs and DERBPs.
2. Bar plot showing the most enriched GO results of DERBPs.
3. Cytoscape shows the co-expression network comprising differentially expressed RBPs from B cells. Edges connect RBP-target gene pairs while nodes represent DEGs. RBPs are displayed in larger font size and deep purple color.
4. Gene ontology enrichment analysis of KEGG pathway of target genes for each RBP from graph C.

**
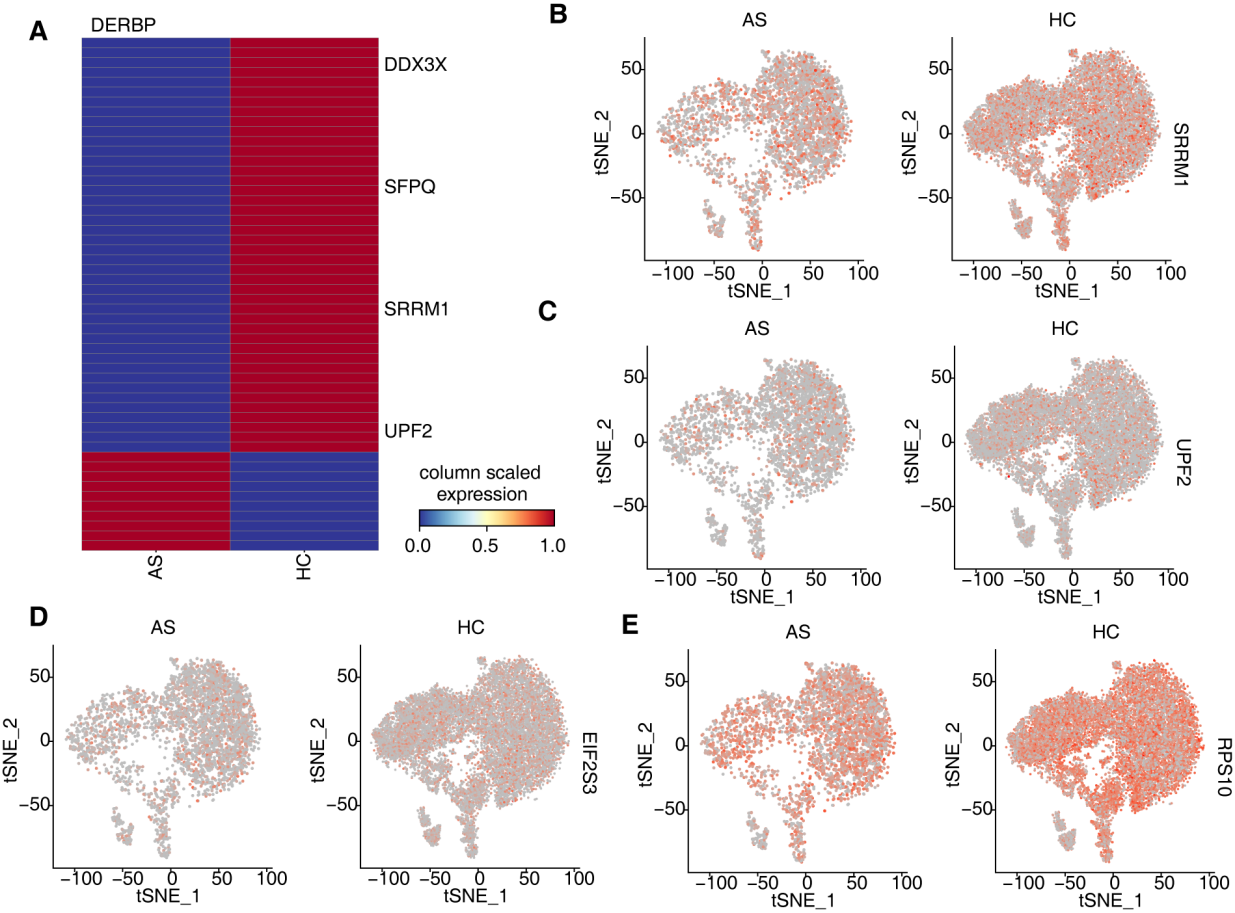
**

**Supplementary Figure S2.scRNA-seq analysis revealed dysregulated RBPs and their regulatory functions in B cells.**

(A) The heatmap showed the expression distribution of DERBPs in different samples in B cells.

(B-E) Gene expression level of UPF2,SRRM1,RPS10,EIF2S3 were represented in the UMAP plot split by different sample groups.

**
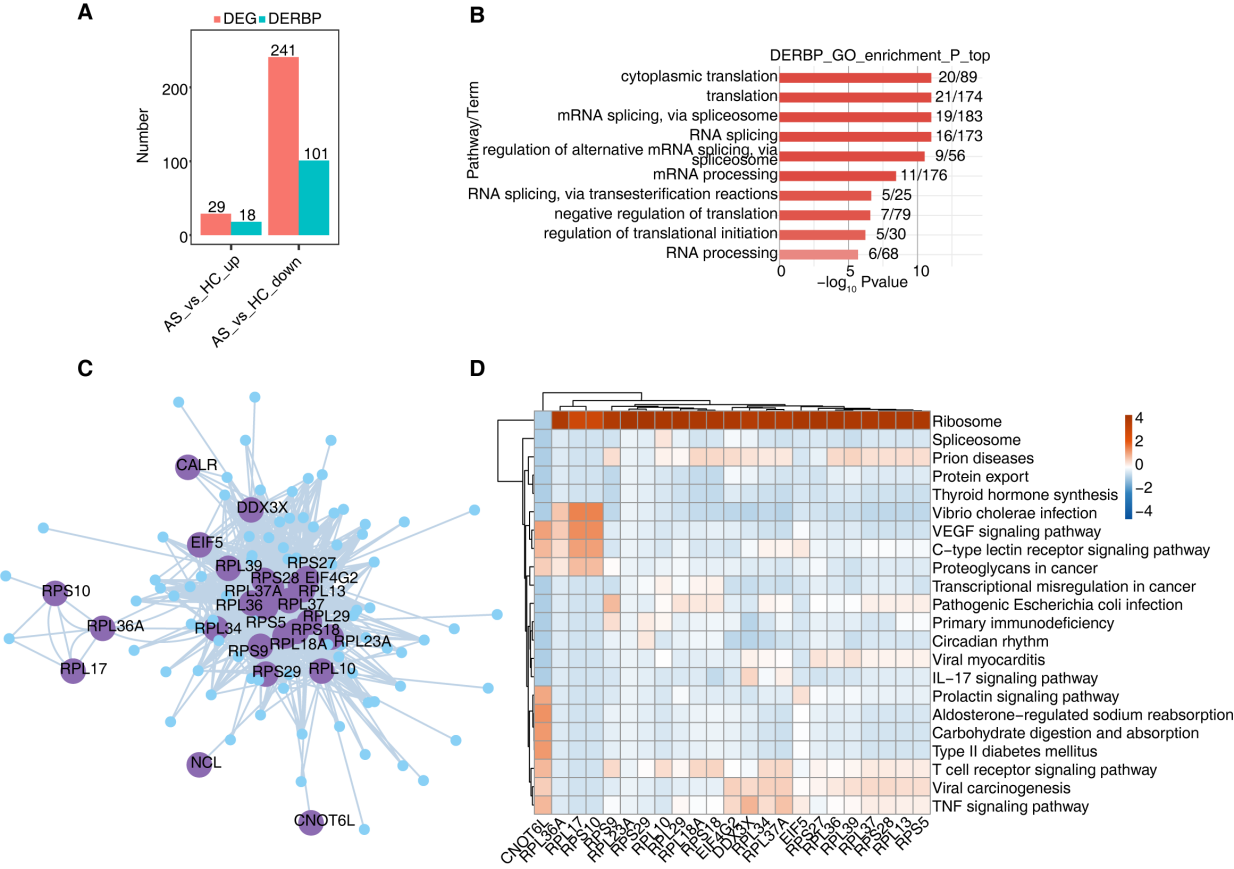
**

**Supplementary Figure 3. ScRNA-seq analysis revealed specific RBPs regulatory module in CD8^+^ T cells.**

1. Bar graphs show the number of DEGs and DERBPs.
2. Bar plot showing the most enriched GO results of DERBPs.
3. Cytoscape shows the co-expression network comprising differentially expressed RBPs from CD8^+^T cells. Edges connect RBP-target gene pairs while nodes represent DEGs. RBPs are displayed in larger font size and deep purple color.
4. Gene ontology enrichment analysis of KEGG pathway of target genes for each RBP from graph C.

**
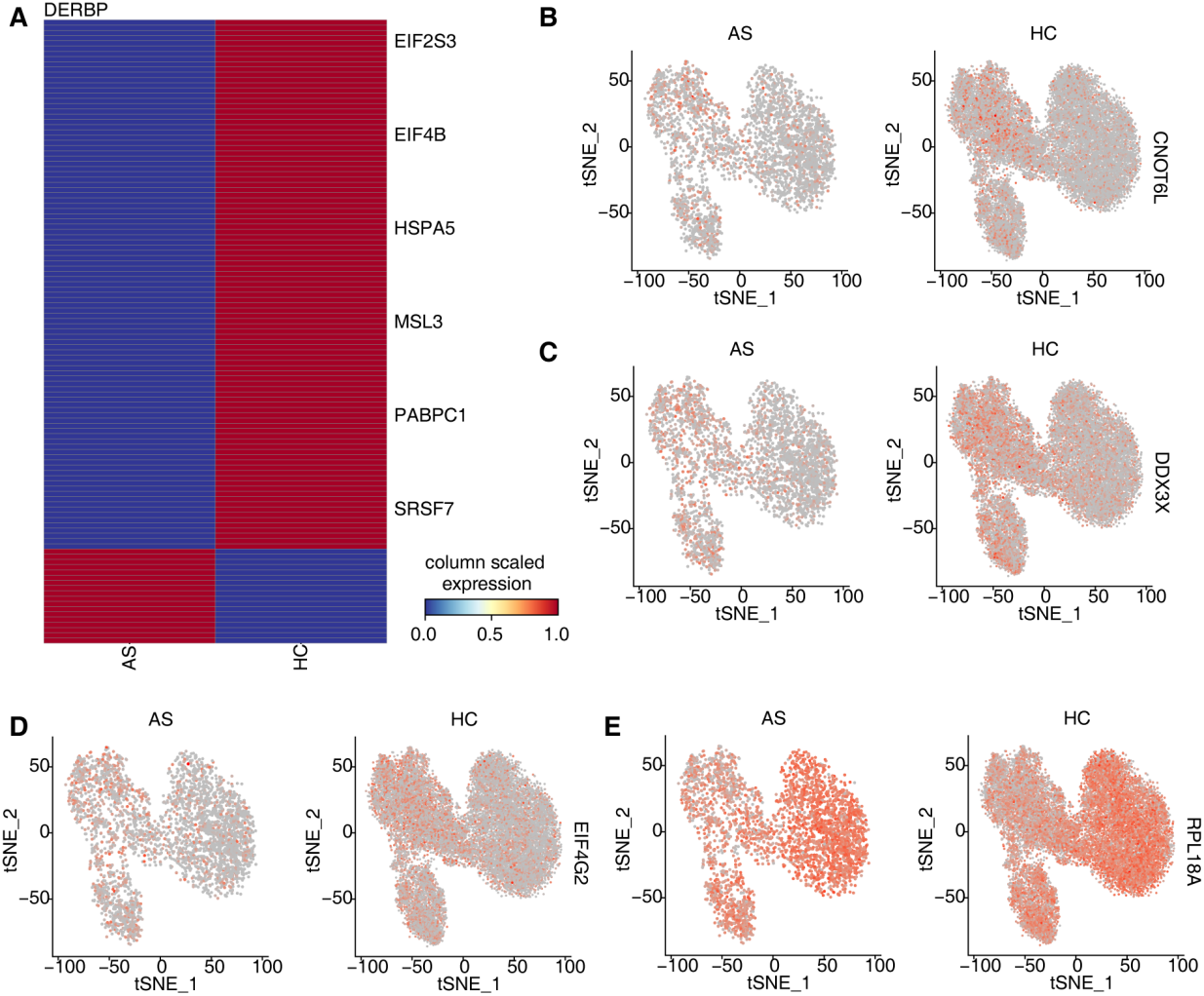
**

**Supplementary Figure S3.scRNA-seq analysis revealed specific RBPs regulatory module in CD8^+^ T cells.**

1. The heatmap showed the expression distribution of DERBPs in different samples in CD8^+^ T cell.

(B-E) Gene expression level of CNOT6L,DDX3X,EIF4G2,RPL18A were represented in the UMAP plot split by different sample groups.

**
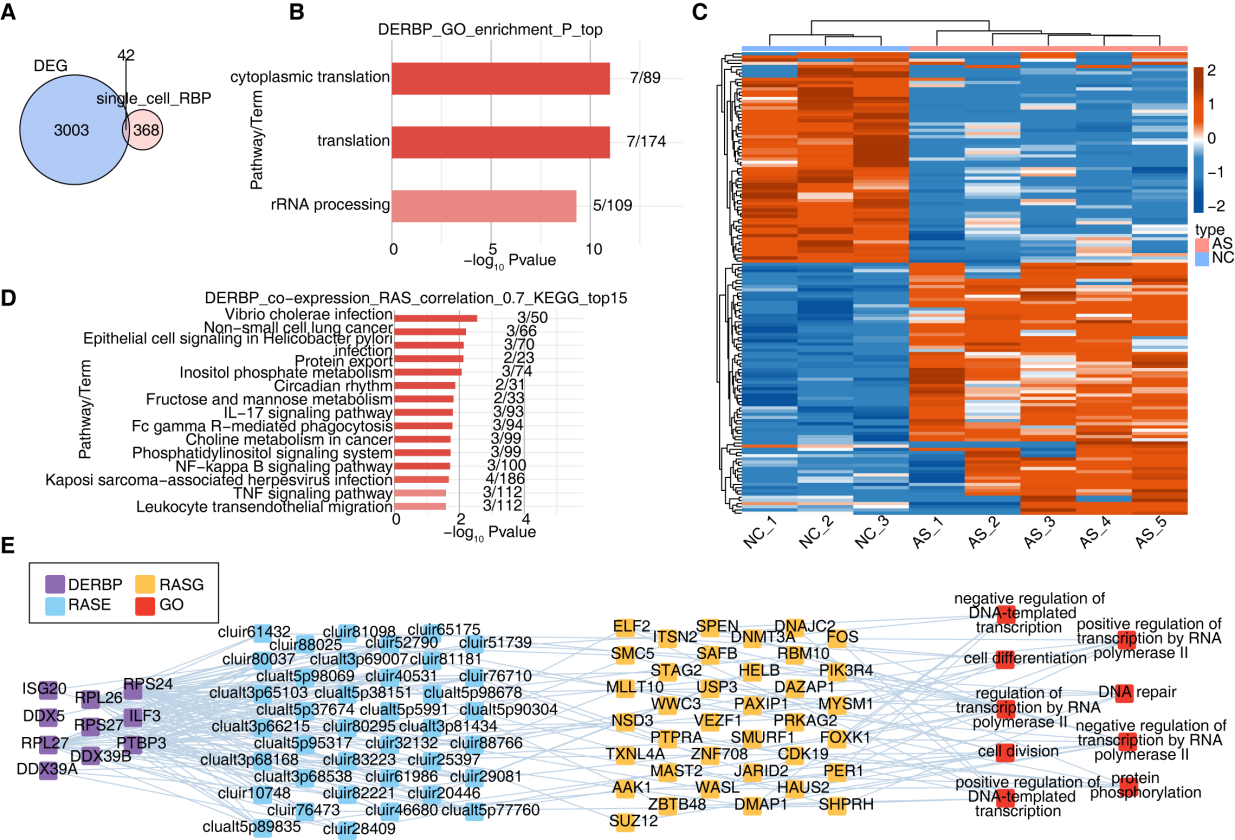
**

**Supplementary Figure 4.Integration with bulk RNA-seq reveals that extensive alternative splicing regulation in ankylosing spondylitis may be related to the abnormal regulation of RBPs.**

1. The Veen shows the number of DERBPs between the DEG and DERBP of single cell.
2. Bar plot showing the most enriched GO results of DERBPs.
3. The Heatmap showing the splicing ratio of specific RAS (PSAR ≥ 50%) in the AS vs NC group.
4. Bar plot showing the top 15 most enriched KEGG results of specific DERBP co-expressed by specific RAS.
5. Co-expression analysis of specific DERBP and specific RAS of key GO biological process results.Cutoffs of Pvalue ≤ 0.05 and Pearson coefficient ≥ 0.7 or ≤ -0.7 were applied to identify the co-expression pairs.The network showing the co-expressed GO pathway for specific DERBP and specific RAS.


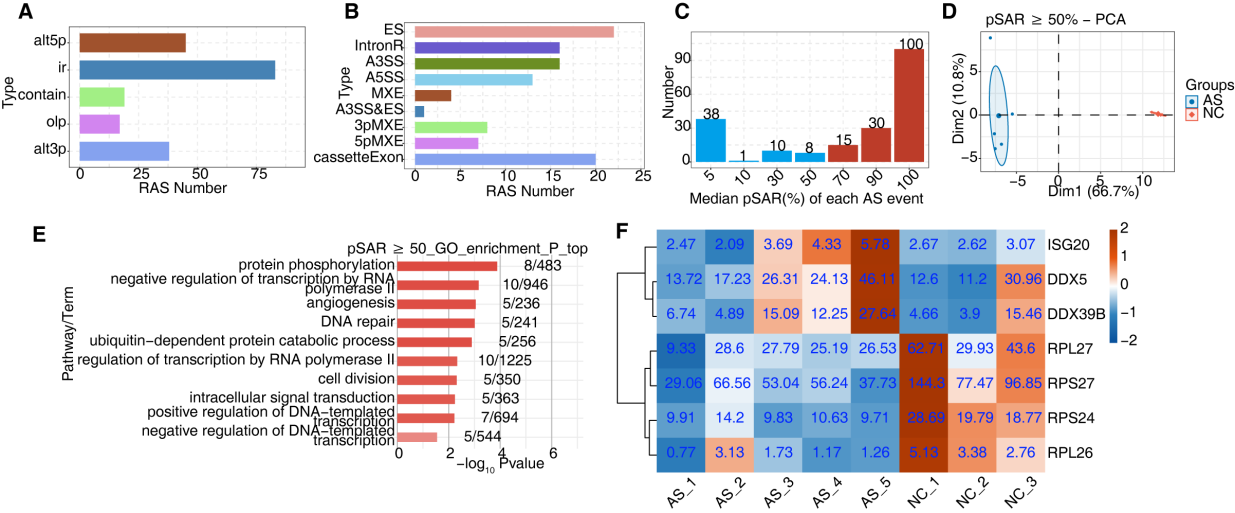


**Supplementary Figure S4.Integration with bulk RNA-seq reveals that extensive alternative splicing regulation in ankylosing spondylitis may be related to the abnormal regulation of RBPs.**

1. Barplot showing number of regulatory RAS detected by Suva in the each group.
2. Splice junction constituting AS events detected by SUVA was annotated to classical AS event types. And the number of each classical AS event types were showed with bar plot.
3. C. Barplot showing RAS with different pSAR. RAS which pSAR (Reads proportion of SUVA AS event) ≥ 50% were labeled.
4. Principal component analysis (PCA) based on RAS of pSAR ≥ 50%. The ellipse for each group is the confidence ellipse.
5. The bar plot exhibiting the most enriched GO biological process results of specific RAS (pSAR≥ 50%) genes in the two groups.
6. The heatmap diagram showing the expression profile of specific DERBP in the two comparison groups.
